# From patient voices to policy: Data analytics reveals patterns in Ontario’s hospital feedback

**DOI:** 10.1371/journal.pdig.0000739

**Published:** 2026-02-05

**Authors:** Pourya Momtaz, Mohammad Noaeen, Konrad Samsel, Neil Seeman, Robert Cribb, Syed Ishtiaque Ahmed, Amol Verma, Dionne M. Aleman, Zahra Shakeri

**Affiliations:** 1 Institute of Health Policy, Management and Evaluation, Dalla Lana School of Public Health, University of Toronto, Toronto, Ontario, Canada; 2 Massey College, University of Toronto, Toronto, Ontario, Canada; 3 Investigative Journalism Bureau, University of Toronto, Toronto, Ontario, Canada; 4 Department of Computer Science, University of Toronto, Toronto, Ontario, Canada; 5 St. Michael’s Hospital, Unity Health Toronto, Toronto, Ontario, Canada; 6 Department of Mechanical & Industrial Engineering, University of Toronto, Toronto, Ontario, Canada; 7 Schwartz Reisman Institute, University of Toronto, Toronto, Ontario, Canada; 8 Faculty of Information, University of Toronto, Toronto, Ontario, Canada; Shahid Beheshti University of Medical Sciences School of Dentistry, IRAN, ISLAMIC REPUBLIC OF

## Abstract

Patient satisfaction is a central measure of high-performing healthcare systems, yet real-world evaluations at scale remain challenging. In this study, we analyzed 122,194 de-identified patient reviews from 45 Ontario general hospitals between January 2015 and July 2022. We applied a natural language processing (NLP) pipeline using a clinical named entity recognition (NER) model fine-tuned on biomedical literature to extract references to diseases, symptoms, and medical procedures from patient reviews. Geospatial analysis was conducted to examine sentiment patterns based on regional census data related to low-income status and visible-minority composition. Our primary objective was to investigate how the COVID-19 pandemic influenced patient satisfaction trends, with a specific focus on clinical units and hospitals serving marginalized populations. We assessed changes in the proportion of positive comments across time periods and socioeconomic groups using multivariate logistic regression.

Our findings show that over 80% of the hospitals studied had fewer than 50% positive reviews, highlighting possible systemic gaps in patient needs. Interestingly, the proportion of negative reviews decreased during the COVID-19 pandemic, suggesting possible changes in patient expectations or increased appreciation for healthcare workers. However, certain units, such as dentistry and radiology, experienced more negative ratings as a proportion of their total reviews. ‘Anxiety’ emerged as a recurrent concern in negative reviews, especially during the start of the pandemic, pointing to the growing awareness of mental health needs. Based on our geospatial analysis, hospitals located in regions with higher percentages of visible minority and low-income populations initially saw higher positive review proportions before COVID-19, but this trend reversed after 2020. Our statistical models confirmed that these shifts were significant, particularly for low-income-serving hospitals. Collectively, these results demonstrate how large-scale unstructured data can identify fundamental drivers of patient satisfaction, while underscoring the urgent need for adaptive strategies to address anxiety and combat systemic inequities.

## Author summary

Understanding what patients think and feel about hospital care can lead to better health services and outcomes. We analyzed more than 120,000 patient reviews from 45 Ontario hospitals between 2015 and 2022. Our study combined text processing techniques to identify key concerns related to factors such as anxiety, billing difficulties, and interactions with staff. We also compared patient experiences before and during the COVID-19 pandemic, uncovering a drop in negative reviews and a rise in positive reviews. A particularly revealing finding was that hospitals located in regions with higher percentages of residents who are visible minorities and low-income groups received more positive feedback before the pandemic, but this trend reversed after the start of the pandemic. These patterns hint at deeper systemic issues, especially during times of crisis. By exploring the main drivers of satisfaction and dissatisfaction, our work highlights the need for healthcare services that prioritize kindness, clear communication, efficient operations, and equitable access for all. Lessons from this research could guide targeted improvements, ensuring that every patient, regardless of background or income, receives the compassionate and timely care they deserve. Our hope is that policymakers, hospital administrators, and community advocates will use these findings to shape policies that improve patient trust and well-being.

## Introduction

Patient-centeredness is essential for delivering high-quality healthcare, focusing on understanding patients’ perspectives, and ensuring safe and effective services [1–3]. Healthcare organizations are increasingly adopting patient-centric models, recognizing that patient satisfaction directly influences treatment adherence, reduces preventable errors, lowers staff turnover, decreases hospital readmissions, and enhances overall patient engagement and health outcomes [4–7]. The World Health Organization emphasizes the strong link between patient satisfaction and improved treatment adherence and health outcomes [6,7]. However, some studies indicate that an excessive focus on patient satisfaction might compromise clinical quality by prioritizing patient preferences over evidence-based practices [8]. This conflicting evidence demonstrates the need for further research, especially in outpatient primary care where data is limited and inconsistent.

A U.S.-based study of acute care hospitals found that those with high adherence to clinical guidelines also achieved better patient experience scores [9]. Interestingly, hospitals with the lowest risk-adjusted mortality rates for acute myocardial infarction had the highest patient experience scores [10]. These findings suggest that prioritizing patient experience does not necessarily undermine clinical quality. Further studies support this, showing that hospitals that excel in patient experience also report lower mortality and readmission rates and better adherence to surgical process measures [9,11,12]. This trend is consistent across various settings, including ambulatory care [11,13–15].

In Canada, focusing on patient experiences and patient-centered care is critical given the country’s health system performance challenges compared to other high-income nations. A 2019 Commonwealth Fund study ranked Canada last among 11 high-income countries for timeliness and efficiency of care [16]. A tragic illustration of these systemic delays comes from a recent case where a patient in Montreal, after waiting six hours for care and ultimately leaving the emergency department, died of an aneurysm [17]. With only 127 family physicians or nurse practitioners per 100,000 Canadians as of 2023 [18], the shortage of primary healthcare providers has led to increased hospital visits during health crises, potentially overwhelming the system [19]. Accessibility is further limited, with only 41% of Canadians able to see a healthcare provider the same or on the next day when needed [20], and even lower rates among economically disadvantaged populations [21], pointing to the need for equitable access to health services.

These accessibility issues significantly impact patient outcomes and experiences. In the 2021-2022 fiscal year, 14.6% of mental health patients in Ontario (Canada) had at least three hospital stays within a year, up from around 0.2% in 2017 [22]. Further compounding this burden, Canadians in the lowest income bracket are three to four times more likely than those in the highest income bracket to report poor or fair mental health [23]. This socioeconomic gradient highlights the disproportionate effects on vulnerable groups, including those with mental health conditions and lower socioeconomic status.

The COVID-19 pandemic exposed and intensified weaknesses in Canada’s healthcare system, such as provider shortages, accessibility issues, and disparities in patient outcomes [24]. Hospital overcrowding, staff shortages, and supply chain disruptions strained resources, with COVID-19 cases and deaths peaking in January 2022 [25]. The pandemic showed the need for resilient healthcare systems and patient-centered care, especially for those with chronic conditions or compromised immune systems [26,27]. Moreover, the mass departure of nursing professionals during the pandemic exacerbated healthcare access issues [28–30]. Unfortunately, even after the direct burden of the COVID-19 had passed, reports indicated continued challenges with long wait times and understaffed facilities [31], emphasizing the need for policy reform.

Prior research has effectively used natural language processing (NLP) and machine learning (ML) to analyze patient reviews, automating tasks such as topic classification and sentiment analysis [32–41]. However, most studies focus on online reviews collected from social media platforms or patient forums rather than from institutional sources. Moreover, there remains a gap in understanding the geographical disparities in patient sentiment, and how the socioeconomic status or marginalization of hospital catchments may relate to healthcare experiences.

In response to these gaps, our study applies NLP and other statistical approaches to identify factors that are associated with patient satisfaction. Our dataset includes 122,194 de-identified patient reviews from 45 out of Ontario’s 47 general hospitals. These reviews, collected between January 2015 and July 2022, offer the opportunity to examine how the COVID-19 pandemic shaped overall trends in patient satisfaction and explore possible differences in these findings when stratifying by clinical unit or catchment demographics. To our knowledge, this is the first study to apply these methods to a review dataset originating directly from multiple healthcare institutions, providing insights into pandemic-era patient experience and health equity within a publicly funded healthcare system.

## Methods

### Dataset

The dataset used in this analysis included 122,194 de-identified patient reviews collected between January 2015 and July 2022 from 45 Ontario hospitals. These reviews were compiled by the US-based National Research Corporation (NRC) and obtained through freedom of information requests by the Investigative Journalism Bureau (IJB) at the University of Toronto. Patient reviews were accompanied by unstructured metadata, including the experience date, hospital name, and hospital ward. Each comment’s sentiment classification was pre-assigned by NRC with one of the following labels: positive, neutral, negative, or mixed (positive/negative). These labels were derived from the patient’s quantitative survey ratings associated with each comment and were utilized as the ground truth for this study and we did not apply an additional automated sentiment model. We excluded comments without corresponding unit names (n = 38,929), resulting in a final analytic sample of 83,265 reviews for unit-level analyses.

### Data labelling and pre-processing

A single-labeler approach was used to identify key themes related to each comment, such as transportation, access, and care coordination. Each review could be tagged with multiple themes, and a full list of thematic labels is included in S2 Table.

Next, patient review comments were pre-processed prior to NER classification, and included tokenization, lemmatization, and removal of non-informative tokens. Misspellings were corrected by mapping to the nearest word in a reference vocabulary. Experience dates were manually extracted and normalized to a consistent JavaScript Date format to support downstream visualization tools. Sentiment valence strings and satisfaction themes were harmonized using rule-based normalization (e.g., lowercasing and lookup tables) [42].

To resolve inconsistent hospital unit labels, we implemented a multi-step process by first defining a list of 27 general unit names (see S1 Table), and then mapping all unit labels in the dataset to these standardized categories using prompt engineering with Claude 3 LLM and manual verification. We used structured prompts to map raw unit labels to standardized unit categories, followed by manual verification. Obvious labels such as “Medical Assessment (MAU)” were directly mapped to “Medical Assessment Unit”, while abbreviations such as “H2C-Neph” were linked to “Nephrology Unit”. Ambiguous codes (e.g., “G4D”) were manually resolved using floor-plan references, and unclear entries (e.g., “YC2BC”) were tentatively categorized based on pattern similarity. Null or unrecognized units were labeled as “Unknown” and excluded from unit-level analyses; this affected 38,929 reviews.

### Clinical named entity recognition

To extract clinically meaningful entities, we used the en_ner_bc5cdr_md model from the scispaCy library [43]. This spaCy-compatible model was trained on the BC5CDR corpus, which includes PubMed abstracts annotated for disease and chemical mentions. It identifies two entity types (DISEASE and CHEMICAL) and achieves an F1 score of approximately 0.86 on BC5CDR datasets [44].

The model returns each recognized entity with its text span and label (e.g., DISEASE or CHEMICAL). We kept entities returned by the model and performed post-processing to support frequency analysis. To capture context and granular meaning, we counted each entity mention separately, even if overlapped (e.g., both “fever” and “acute fever” were counted). We focused our analysis on clinically relevant disease entities (e.g., “pneumonia,” “cancer”) and chemical substances (e.g., specific medications). These extracted spans were aggregated to compute mention frequencies across all reviews.

### Geospatial analysis by hospital catchment

We examined demographic factors using the *HealthyPlan.City* platform [45], developed by the Canadian urban environmental health research consortium (CANUE) [46,47], which integrates environmental indicators at the dissemination-block level with 2021 Canadian Census data across more than 125 Canadian cities [48]. For each hospital, we calculated a population-weighted average of visible-minority and low-income percentages across all dissemination blocks intersecting a geographic radius from the hospital’s location. While a 5 km radius was used by default, a smaller radius of 3 km was applied when two hospitals were located within 10 km of one another. This adjustment helped reduce overlap between catchment areas and ensured clearer attribution of surrounding neighborhood characteristics to each hospital. Hospitals were classified as serving higher visible-minority populations if ≥40% of nearby residents identified as visible minorities, and higher low-income populations if ≥10% of residents fell below the after-tax low-income cut-off (LIM-AT), as defined by Statistics Canada [49]. These population characteristics were computed as population-weighted averages across dissemination blocks within the designated radius. For the geospatial demographic analysis, data from 2015 were excluded due to low sample volume (*n* = 541) compared to subsequent years to ensure robust annual comparisons starting from 2016.

### COVID-19 period temporal segmentation

‘COVID-19 period’ reviews were defined as those dated from January 2020 to July 2022. This range aligns with the World Health Organization’s emergency designation timeline [50,51], enabling consistent comparison of patient sentiment trends before and during the pandemic.

### Statistical analysis

To evaluate differences in sentiment trends, we fitted multiple logistic regression models, each focusing on specific variable groupings (e.g., hospital units, thematic codes, demographic designations). The dependent variable across all models was binary sentiment polarity (1 = Positive, 0 = Negative). For binary sentiment models, we restricted analyses to reviews labeled positive or negative, excluding neutral and mixed (positive/negative) labels. Independent variables in these models included review year, hospital unit, thematic codes, and binary indicators for COVID-19 period, minority-serving hospitals, and low-income hospital regions. Interaction terms (e.g., CovidPeriod × Minority) were incorporated where applicable to assess whether temporal sentiment shifts differed across hospital and population groups.

Model significance was assessed using maximum likelihood estimation via the statsmodels library [52]. For each interaction model, we performed a likelihood ratio test comparing full and reduced models to determine whether interaction terms contributed significantly to model fit. Standardized z-scores, p-values, and 95% confidence intervals were reported for each coefficient.

We also conducted post-hoc statistical tests, including two-proportion z-tests, to compare sentiment proportions across pre-COVID and COVID periods. Chi-square test was used to assess temporal trends across years. All statistical analyses were conducted in Python using the following data analysis libraries: pandas(v2.2.2), scipy(v1.15.3), and statsmodels(v0.14.15). Significance was defined based on an alpha value of 0.05.

## Results

### Preliminary results

**Fig 1:**
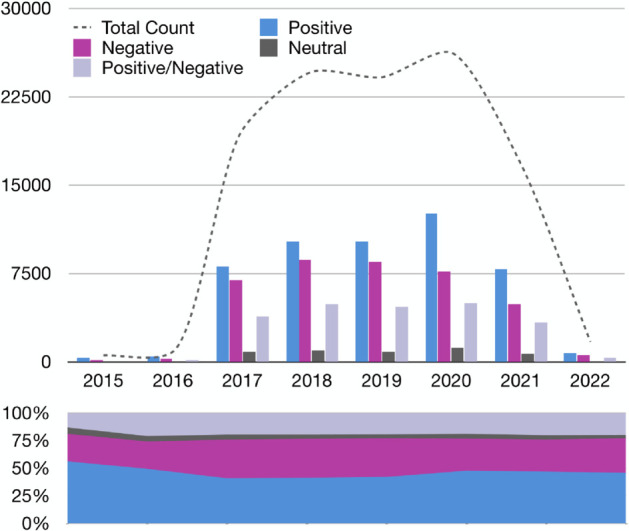
Temporal analysis of review sentiment distribution (2015-July 2022). The upper panel presents a grouped bar chart depicting the absolute frequency of positive, negative, neutral, and mixed (positive/negative) reviews annually. The lower panel illustrates the relative proportion of each sentiment category as a stacked percentage bar chart. This dual representation reveals both absolute and relative changes in review sentiments over time, signaling trends in patient feedback and potential external influences.

Based on our analysis of 122,194 de-identified patient feedback comments collected between 2015 and 2022 across 45 Ontario hospitals, we examined overall sentiment patterns and shifts over time. Our analysis showed that over 80% of these institutions received under 50% positive reviews, suggesting significant potential to enhance patient satisfaction. The total volume of reviews varied sharply from 2015 to 2022, rising from 541 reviews in 2015 to a peak of 26,243 in 2020 before dropping to 1,708 in 2022 (Fig 1). These fluctuations may reflect shifts in patient engagement through online feedback platforms, combined with an uptick in healthcare discussions during the early pandemic phase. The lower numbers for 2022 could stem from the dataset including reviews only until July of that year.

Fig 1 illustrates trends in sentiments from 2015 to July 2022. Positive reviews were generally the largest category, ranging from 56.19% (2015) to 40.84% (2017), while negative reviews spanned 24.77% to 35.31%. Neutral reviews stayed below 5.73%. Although positive feedback climbed from 2017 to 2020, it slipped from 47.7% in 2020 to 45.8% in 2021. Concurrently, negative feedback rose from 29.11% to 31.4%. This result points to the pandemic’s probable impact on patient satisfaction. Year-by-year, positive sentiment rose through 2020 and then declined slightly in 2021. However, when we aggregated reviews into pre-COVID (2017–2019) versus COVID period (2020–2022), the odds of positive sentiment were higher during the COVID period (OR = 1.34, 95% CI 1.29–1.38). This suggests that sentiment shifts were not uniform across pandemic years. To assess this observation, we conducted a chi-square test comparing the distribution of positive, neutral, and negative reviews across pre-COVID and COVID periods. The test revealed a significant shift ($\chi^{2}$ = 350.84, *p* < 0.0001), indicating that patient sentiment varied meaningfully across these periods. The shift in sentiment during the COVID-19 period was also confirmed through logistic regression and two-proportion z-tests. We fit a logistic regression model using only the COVID period as a binary predictor of review sentiment. The results showed a significant increase in the odds of positive sentiment during COVID-19 (*p* < 0.001), with an estimated 34% higher odds of positive reviews during the pandemic period. The model odds ratio for the COVID period was 1.34 (95% CI: 1.29–1.38), indicating a robust and consistent positive shift. This finding was supported by a two-proportion z-test comparing the fraction of positive reviews in the three years before COVID (2017–2019) versus the three years during the pandemic (2020–2022). The proportion of positive sentiment increased from 54.9% to 62.3%, (i.e., computed among reviews labeled positive or negative, excluding neutral and mixed labels) a statistically significant rise (*Z* = −18.37, *p* < 0.0001). This confirms that the pandemic period was associated with a meaningful upward shift in expressed patient satisfaction.

Word-frequency analysis suggested that staff behaviour, rather than clinical outcomes, drove both favorable and unfavorable reviews. Terms such as ‘nurse’ and ‘staff’ were widespread. Positive responses frequently included words like ‘professional’, ‘helpful’, and ‘caring’, indicating that qualities like competence and empathy form a foundation for trust. Negative reviews often cited ‘waiting’, ‘time’, ‘night’, and ‘hour’, suggesting long waits and logistical delays were a major source of frustration. An elevated mention of ‘doctor’ implied an opportunity to improve interactions with physicians, which could have shaped overall care perceptions. These insights signaled that interpersonal communication and efficient operations were top priorities for boosting satisfaction, mirroring global perspectives on patient-centered care [13].

### Trends in negative and positive reviews

Analysis of patient reviews across different hospital units showed varied patterns in satisfaction. Some units reported more negative sentiments, while others held steady or improved positive ratings. The percentages listed below reflect the share of all positive (or all negative) reviews attributable to each unit in a given year (i.e., unit volume composition), not the within-unit positivity rate. To evaluate whether the pandemic affected sentiment differently across units, we first restricted the dataset to reviews labeled as either ‘Positive’ or ‘Negative’, excluding neutral or mixed responses. We then created a binary sentiment variable (1 = Positive, 0 = Negative) to serve as the outcome for a logistic regression model. The model included the review period (pre-pandemic vs during-pandemic), hospital unit type, and their interaction as predictors. Results showed that the effect of COVID-19 on sentiment varied significantly by unit (*p* = 0.012, likelihood-ratio test), indicating a heterogeneous response to the pandemic across hospital services.

Select results from this model are shown in Table 1. Day Surgery Units saw a significantly greater increase in positive sentiment during COVID (OR = 1.2760, *p* = 0.016), while Dentistry showed a significant decline (OR = 0.4140, *p* = 0.008). Oncology Units experienced a moderate but statistically significant increase in positivity (OR = 1.4569, *p* = 0.008). Emergency Departments remained significantly below average in baseline sentiment, but did not show a COVID-specific shift (*p* = 0.602, see Fig 5). Cardiology Units maintained a generally positive sentiment, with no significant change across the COVID boundary (*p* = 0.438, see Fig 5). The full results of this model, including all unit effects and interaction terms, are reported in S3 Table, along with model statistics and likelihood-ratio test.

**Table 1:** Effect of COVID-19 period on patient sentiment across hospital units: Selected results from logistic regression model with interaction terms.

Unit	Odds ratio (95% CI)	*p*-value
Day Surgery	1.2760 (1.0501–1.5493)	0.016
Dentistry	0.4140 (0.2169–0.7894)	0.008
Oncology	1.4569 (1.1027–1.9251)	0.008
Emergency Department	1.0214 (0.9423–1.1065)	0.602
Cardiology	0.9311 (0.7799–1.1104)	0.438

*Note.* Results shown are a subset of unit-specific interaction effects from a logistic regression model predicting binary sentiment (positive vs. negative) as a function of hospital unit, COVID-period (Jan 2020–Jul 2022), and their interaction. Full results are available in S3 Table. Analytic sample: 83,265 patient reviews from 45 Ontario hospitals.

The emergency unit reported a rise in negative reviews, moving from 36.1% in 2017 to 44.3% in 2021. This aligned with recent data showing Ontario’s ‘hallway health care’ challenges were worse than ever, as crowded hallways and bed shortages persisted [53]. Longer wait times, crowded facilities, and understaffing might explain this spike. Reports from Ontario’s Ministry of Health have noted that many emergency departments face staff shortages, which could have amplified patient dissatisfaction. Meanwhile, the maternity and pediatric units maintained low negative-review percentages. The maternity unit’s share of negative reviews shrank from 1.8% in 2017 to 0.3% in 2021, and the pediatric unit’s share went down from 4.1% to 1.1%. The medical/surgical unit also showed consistently lower negative reviews, varying from 4.1% in 2017 to 2.5% in 2021. This stability might reflect readiness for pandemic-related demands. Some local reports attribute this resilience to well-defined protocols that helped staff respond to workload surges without compromising care quality.

Although the emergency unit struggled with increased negative perceptions, it also showed a rise in positive reviews, climbing from 30.6% in 2017 to 44.7% in 2021. This shift may point to effective problem-solving measures in emergency care. In contrast, the maternity unit faced a drop in positive reviews, going from 3.6% in 2017 to 0.9% in 2021, while the pediatric unit’s positive reviews dropped from 5.8% to 2.5%. COVID-19 restrictions, such as limited visitors and face coverings, may have affected maternity and pediatric feedback [54–56]. These findings suggested a complex balance between providing safe care and addressing patient preferences, especially in units focused on women’s and children’s health.

### Key clinical entities in patient reviews

**Fig 2:**
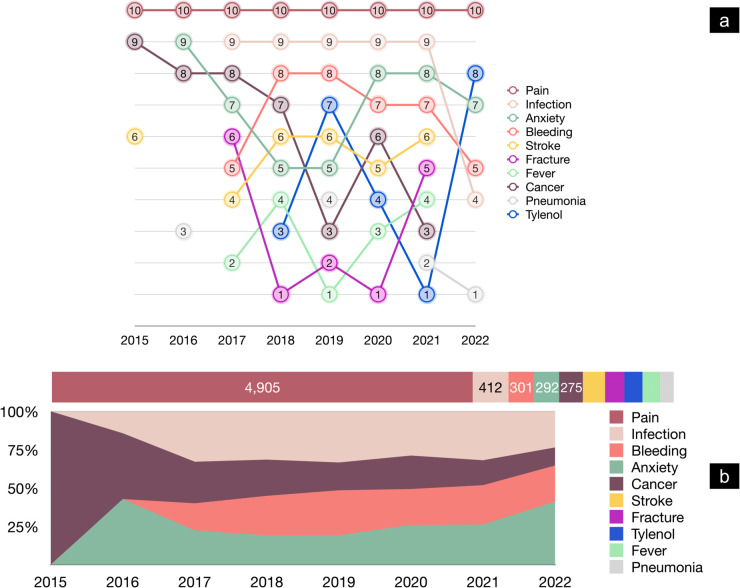
Common entities in negative patient reviews (2015-July 2022). (a) Bump chart showing annual ranking trends of frequently mentioned clinical entities, with 10 representing the most frequent. (b) Stacked bar chart (top) depicting raw counts and normalized area chart (bottom) showing the relative prevalence of the most frequent entities over time.

Named entity recognition (NER) uncovered trends in frequently mentioned clinical topics in negative reviews. ‘Pain’ stood out as the most frequent term (Fig 2a), totaling 4,905 mentions (Fig 2b). Patients are often asked to describe their pain, which may drive these high counts.

‘Infection’ was second most mentioned from 2017 to 2020 before dropping to seventh in 2022, indicating changing patient concerns over time. ‘Anxiety’ rose from being unranked in 2015 to among the top five from 2020 onward, reflecting a sharper focus on mental health in hospital reviews during the pandemic. ‘Bleeding’ and ‘stroke’ remained consistent, while ‘cancer’ and ‘fracture’ shifted in importance. These changes may match evolving hospital services or patient demographics. They also emphasize the need for flexible care models that respond to new demands, such as rising orthopedic cases or advanced cancer treatments.

In negative reviews, leading concerns included ‘pain’, ‘infection’, ‘bleeding’, ‘anxiety’, and ‘stroke’. In positive reviews, ‘pain’, ‘cancer’, ‘stroke’, ‘anxiety’, and ‘infection’ took the top spots. The changing positions of these terms between the pre-COVID and COVID-19 periods (Fig 2b) showed that patient priorities shifted under different conditions. Word frequency analysis also revealed that beyond clinical terms, patient satisfaction was linked to staff interactions and efficient service. Positive reviews featured words such as ‘professional’ and ‘caring’, whereas negative reviews included ‘waiting’ and ‘time’. This points to the importance of addressing operational hurdles and ensuring compassionate communication to strengthen patient trust.

### Performance during COVID-19

Our analysis found that the percentage of negative reviews during the COVID-19 period was approximately 6 percentage points lower than during the non-COVID period, alongside a higher percentage of positive reviews. While this shift in sentiment may suggest increased public sympathy toward hospitals during the pandemic [57], or successful adaptations in service delivery, it should be interpreted as a correlation rather than a causal effect. Other possible explanations include a reduction in hospital visits, changes in triage protocols, or the expansion of telehealth services, which may have influenced patient sentiment during this time.

The analysis of key themes showed that during the COVID-19 period, ‘positive recognition’ had the highest percentage of positive reviews (73.18%), while ‘billing/accounting’ had the highest percentage of negative reviews (63.84%). In the non-COVID period, ‘positive recognition’ also had the highest percentage of positive reviews (73.77%), and ‘billing/accounting’ had the highest percentage of negative reviews (68.34%). This result implies that while many patients appreciated staff acknowledgment, billing issues remained a major source of dissatisfaction.

**Fig 3:**
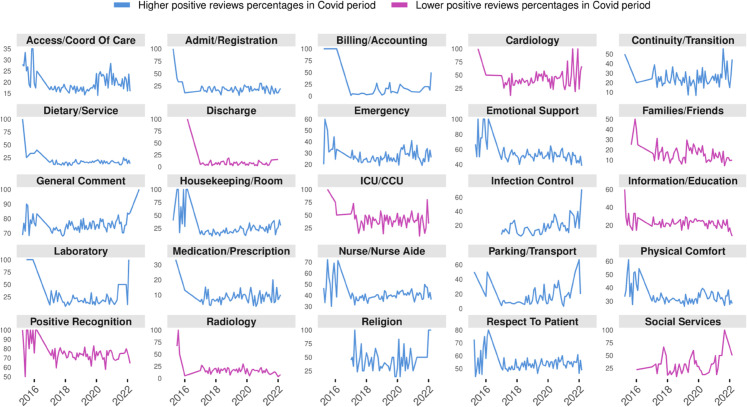
Positive review percentages for key hospital themes before and during COVID-19. Themes like ‘ICU/CCU’, ‘cardiology’, and ‘radiology’ experienced declines, indicating significant challenges during the pandemic. In contrast, other themes showed increased positive reviews, reflecting hospitals’ resilience in enhancing patient experiences amid unprecedented challenges.

Comparisons of positive review percentages (Fig 3) suggested that ‘ICU/CCU’, ‘cardiology’, ‘radiology’, ‘families/friends’, ‘social services’, ‘discharge’, ‘information/education’, and ‘positive recognition’ received fewer positive reviews during COVID-19, suggesting the pandemic’s uneven impact. ICU/CCU and cardiology departments, for instance, were pushed to the limit by surging critical cases, possibly dampening patient satisfaction. Other themes showed higher positive review rates, pointing to the adaptability of some services.

To assess the impact of the COVID-19 pandemic on these patterns, we also used a logistic regression model that included both the themes and a binary indicator of whether the review occurred during the pandemic. We also included an interaction term between the theme and the COVID-19 period, allowing us to test whether the association between a given theme and sentiment changed during COVID.

The results showed that many themes were statistically significant predictors of patient sentiment (*p* < 0.001 for most), confirming their importance in shaping experiences. Moreover, several themes showed a clear shift during the pandemic. For example, while “emotional support” (OR = 0.73, 95%CI:[0.64 − 0.83]) and “respect to patient” (OR = 0.78, 95%CI:[0.71 − 0.87]) remained strong drivers of positivity overall, their sentiment impact weakened slightly during COVID, suggesting increased strain on interpersonal aspects of care. Conversely, themes like “infection prevention and control” (OR = 2.32, 95% CI:[1.59 − 3.38]) and “housekeeping” (OR = 1.28, 95%CI:[1.08 − 1.52]) saw a relative improvement in sentiment during the pandemic, potentially reflecting heightened public awareness or appreciation of these areas.

This statistical analysis supported the idea that not only did sentiment vary by theme, but the meaning and importance of those themes also shifted in response to the unique conditions of the pandemic. A complete summary of the regression model, including all themes and interaction terms, is provided in S4 Table.

**Fig 4:**
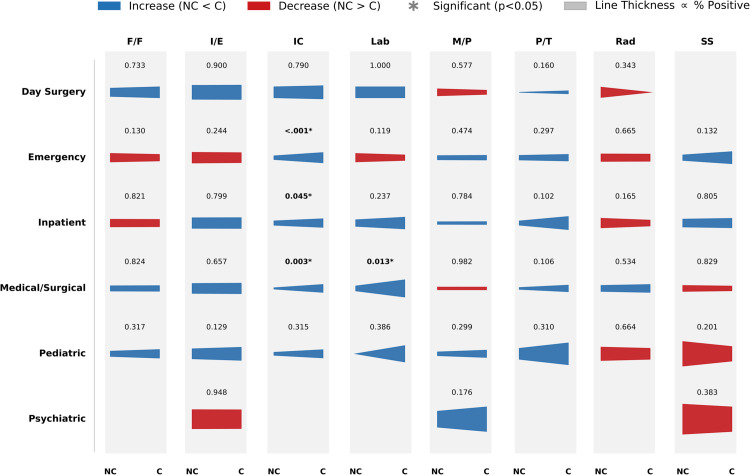
Shifts in positive patient sentiment by hospital unit and theme (Pre-COVID vs. COVID-19). This comet chart visualizes the change in the percentage of positive reviews from the Pre-COVID (NC) period (comet tail) to the COVID-19 (C) period (comet head). On the left axis are hospital units, and along the top axis are key themes: information/education (I/E), medication/prescription (M/P), infection prevention & control (IC), families/friends (F/F), parking/transport (P/T), social services (SS), laboratory (Lab), and radiology (Rad). P-values displayed above each comet were calculated using a Chi-square test of independence comparing the proportion of positive vs. negative reviews between the two periods. An asterisk (*) indicates statistical significance (*p* < 0.05).

Fig 4 details the trajectories of positive sentiment across specific hospital units and themes. Statistical analysis (Chi-square test) revealed significant declines in satisfaction in key areas. Notably, Pediatric units saw a sharp, statistically significant drop in positive sentiment regarding ‘Families/Friends’ (*p* < 0.001), likely reflecting the strain of visitation restrictions on family-centered care. Similarly, Inpatient units experienced significant decreases in positive feedback for ‘Social Services’ and ‘Parking/Transport’ (*p* < 0.001), indicated by the prominent red downward trajectories. In contrast, themes such as ‘Infection Control’ showed upward trends (blue trajectories) across several units (e.g., Emergency, *p* < 0.05), suggesting that despite operational challenges, patients recognized and appreciated the enhanced safety protocols implemented during the pandemic.

**Fig 5:**
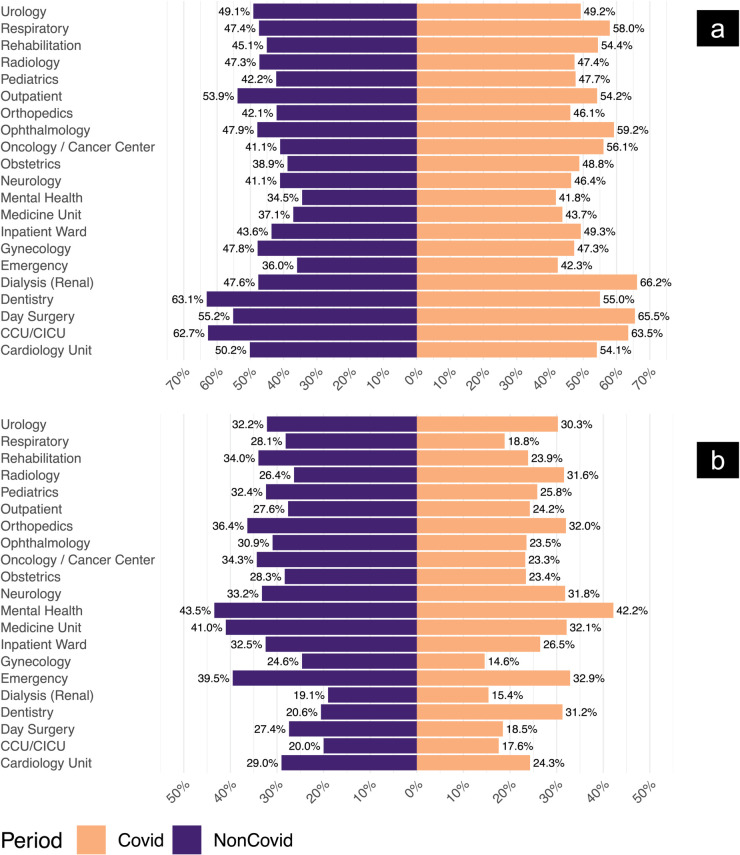
Positive and negative review percentages across different hospital units (COVID-19 vs. non-COVID periods). (a) Percentages of positive reviews. (b) Percentages of negative reviews. This dual representation captures the shifts in patient satisfaction and dissatisfaction across different hospital units due to the pandemic, revealing critical insights into the impacts on healthcare services and areas needing targeted improvements.

Positive review percentages rose for most hospital units during COVID-19 compared to the non-COVID period, with dentistry as a notable exception (31.25% vs. 20.57%). Cardiology and day surgery units saw relatively high positive ratings during COVID-19 (51.47% and 56.01%, respectively) (Fig 5a), which may reflect stronger infection control measures and patient safety protocols [58]. For dentistry and radiology, lower positive reviews (and higher negative ones) may stem from restricted service availability or heightened anxiety about close-contact procedures.

Emergency and inpatient units had lower negative percentages during COVID-19 (32.81% and 26.89%) than before (38.91% and 31.92%), hinting that fewer non-urgent visits or increased public empathy may have contributed to slightly better perceptions (Fig 5b). These results illustrate how crisis responses can shape patient satisfaction [59]. Dentistry and radiology, on the other hand, indicate unmet needs that demand targeted solutions. A recent multicenter survey reported similar diverging trends, reinforcing the idea that some units handled acute pressure better than others [60].

### Equity, Diversity, and Inclusion (EDI) analysis

**Fig 6:**
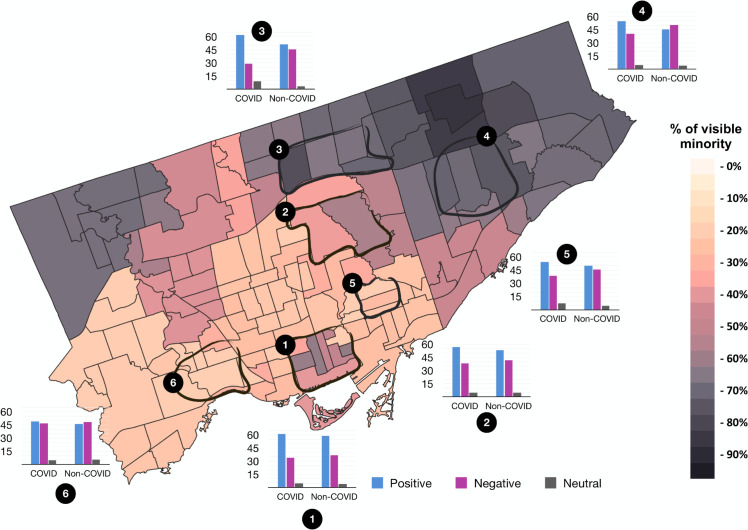
Visible minority demographics and hospital review sentiments in Toronto. A heatmap of visible minority percentages across Toronto’s six hospital regions, alongside bar charts depicting patient review sentiments. Regions 1-4 exhibit >40% visible minority populations, while regions 5-6 show <40%. The base map data were obtained from the University of Toronto Maps and Data Library under a CC-BY license. The map was created in QGIS, with final styling completed for the manuscript

Fig 6, generated with QGIS from shapefiles downloaded from the University of Toronto Maps Library, shows neighborhood boundaries used in our demographic sentiment analysis. Visible minority percentages ranged from <20% to >90%, yet high negative review rates did not always align with areas of higher minority density, indicating that other elements like socioeconomic conditions or local health resources may have been pivotal. Specifically non-COVID period reviews consistently received more negative reviews from the COVID period, suggesting persistent obstacles in everyday healthcare. Positive review rates varied by region, which can reflect possible differences in staffing or local health policies.

**Fig 7:**
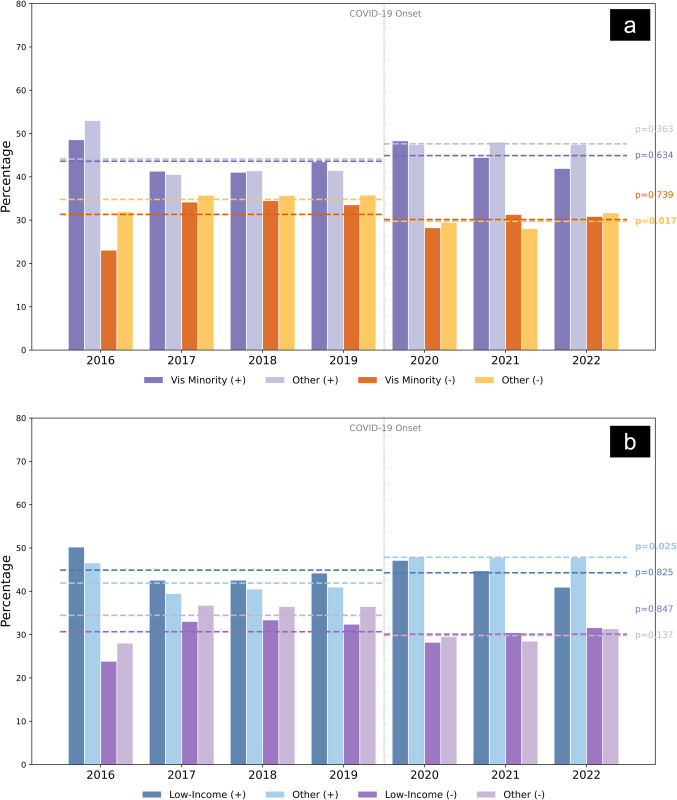
Comparison of mean sentiment before and during COVID-19 (2016-2022). Grouped bar charts showing the percentage of positive (+) and negative (-) comments for (a) visible minority groups and (b) low-income groups compared to others. Dashed lines represent the mean sentiment for the Pre-COVID (2016–2019) and COVID period (2020–2022) periods. P-values (t-test) indicate the significance of the change in means between the two periods, where *ns* denotes not significant (*p* > 0.05), while (*p* < 0.05) indicates statistical significance.

**Visible minority analysis: Pandemic exacerbates existing inequalities–** Analysis of annual sentiment averages reveals a widening gap during the pandemic. In the pre-COVID period (2016–2019), hospitals in high-minority areas maintained a positive review average of 43.6%. During the COVID-19 period (2020-2022), this average remained statistically unchanged (Mean = 44.9%, p=0.63). In contrast, hospitals in low-minority (‘Other’) areas saw a significant increase in positive sentiment, rising from a pre-COVID baseline of 41.6% to 47.6% during the pandemic (*p* = 0.015). This indicates that the general uplift in public gratitude observed in other regions was not equally felt in high-minority catchments (Fig 7a).

To statistically examine this trend, we conducted a logistic regression using hospital minority status, COVID period, and their interaction as predictors of sentiment polarity. The model confirmed a strong overall increase in positive sentiment during the COVID period (*p* < 0.001). The interaction between COVID period and minority-serving hospitals was marginally significant (*p* = 0.053), suggesting a subtle but meaningful decline in sentiment among hospitals serving higher-minority populations during the pandemic. A detailed summary of the model is presented in S5 Table.

**Low-income analysis: Economic vulnerability amplifies healthcare disparities–** A similar pattern of stagnation was observed in low-income regions. While hospitals in higher-income (‘Other’) catchments saw their average positive reviews rise markedly from 41.9% (Pre-COVID) to 47.9% (COVID period) (p = 0.012), hospitals in low-income regions showed no statistically significant change, moving from a pre-COVID average of 44.9% to a COVID period average of 44.3% (p = 0.83). Furthermore, while higher-income areas benefited from a significant reduction in negative reviews (dropping from 34.4% to 29.8%, p = 0.046), low-income areas saw no such relief, with negative review proportions remaining stable (p = 0.86). As shown in Fig 7b, these trends highlight that economically vulnerable communities did not share in the broader improvements in hospital sentiment observed during the pandemic.

To statistically validate this reversal, we ran a logistic regression model that included low-income status, the COVID period, and their interaction. Prior to the pandemic, hospitals serving low-income populations were significantly more likely to receive positive sentiment (*p* < 0.001). However, the interaction term revealed a significant decline in positive sentiment during COVID (*p* < 0.001), confirming a regression of sentiment in these hospitals as the pandemic progressed. This shift aligns with our observed trends and supports the interpretation that economically vulnerable communities faced disproportionate strain during public health crises. A detailed summary of the model is included in S6 Table.

## Discussion

### Key observations

Below we present the main findings from our analysis, drawing attention to patterns that could reshape healthcare practices and policies.

### Patient satisfaction as a launchpad for change

In today’s digital age, over 70% of patients research hospital reviews before deciding where to receive care, a significant increase from just 25% in 2013 [61], demonstrating the growing impact of online feedback on patient decisions. However, despite this trend, a recent report reveals that Ontario hospitals, while collecting substantial patient feedback, kept the findings confidential, leaving patient pleas for improvements unheard [60]. The finding that over 80% of hospitals had less than 50% positive reviews signals a pressing need for major overhaul in our healthcare system, aligning with Canada’s last-place ranking among 11 high-income nations in timeliness and efficiency of care [62], indicating a critical need for broad reform. The COVID-19 period was associated with measurable shifts in patient sentiment. While our aggregated models show higher overall odds of positive sentiment during COVID compared to pre-COVID, year-by-year patterns suggest that sentiment fluctuated across pandemic years, with some areas showing declines in positivity. From 2020 onwards, the gap between negative and positive reviews narrowed, suggesting a growing sense of dissatisfaction. This decline stems from intense pressure on healthcare resources, rapid adoption of new safety measures, and rising patient anxiety during an unpredictable global crisis [24]. Nevertheless, the high proportion of negative reviews may partly reflect self-selection bias, where patients who experience dissatisfaction are more inclined to leave feedback. Furthermore, the data collection methods used by hospitals or external review platforms could amplify this effect. However, the consistent pattern of concerns across multiple hospitals suggests that significant systemic issues remain unaddressed, and the urgency of such issues is not diminished by potential sampling biases.

### Impact of staff behaviour on patient perceptions

Our analysis shows that human interaction is central in healthcare. Positive reviews often mentioned staff attributes such as ‘professionalism’, ‘helpfulness’, and ‘attentiveness’, aligning with research that calls attention to communication, empathy, and respectful treatment as key factors in patient experiences and outcomes. Negative reviews, in contrast, frequently pointed out long wait times, which is consistent with studies showing that excessive waits can lower satisfaction [63,64].

These patterns underline the importance of well-rounded training initiatives that target clinical skills, interpersonal engagement, emotional intelligence, and patient-focused dialogue. Creating a culture of compassion and respect can boost patient satisfaction and overall well-being [65–67]. The frequent mention of ‘doctor’ in negative reviews is especially concerning, suggesting issues such as high workloads, time constraints, or insufficient interpersonal training. Further investigation is warranted, possibly through targeted surveys, focus groups, and robust feedback analysis. We advocate for holistic training, more inclusive workplace cultures, effective strategies to shorten wait times [68,69], and real-time monitoring of patient feedback to address these critical issues.

### Challenges and adaptations during the COVID-19 pandemic

The COVID-19 pandemic introduced extraordinary hurdles for healthcare systems. Our analysis showed evidence of changes in patient satisfaction trends during this period. Negative reviews often included ‘anxiety’ and ‘infection’, reflecting the pandemic’s mental and physical toll on patients [24]. Surprisingly, the share of negative reviews dropped while positive reviews rose during COVID-19 compared to pre-pandemic times. This might be attributed to increased patient empathy for overburdened hospitals, heightened focus on infection control, and adjusted patient expectations favoring basic care delivery over non-essential aspects [70,71].

Still, some hospital units showed disparities. The emergency unit consistently received high negative feedback, while the day surgery unit maintained higher positive ratings. This difference hints that certain services were prioritized, possibly resulting in uneven patient experiences across departments.

### Drivers of patient satisfaction and dissatisfaction

Our analysis uncovered major factors that shape satisfaction or dissatisfaction. ‘Positive recognition’ consistently drove satisfaction, garnering the highest proportion of positive feedback before and during COVID-19. This finding reflects the value of recognition and gratitude in healthcare. Problems with ‘billing and accounting’, on the other hand, were a leading cause of dissatisfaction, with 68.34% negative reviews in the non-COVID period and 63.84% negative reviews in the COVID period, which indicates a need for clearer billing procedures and improved communication around financial matters.

We discovered that high costs for ancillary items and prescriptions contributed significantly to dissatisfaction, a phenomenon also noted in Canadian studies of out-of-pocket drug expenses [72]. Some patients also pay extra to skip long queues, intensifying their financial burden and unhappiness [72,73]. These data call for more transparent billing practices and action on healthcare affordability.

Notably, certain themes such as ‘ICU/CCU’, ‘cardiology’, ‘radiology’, ‘families/friends’, ‘social services’, ‘discharge’, ‘information/education’, and ‘positive recognition’ saw lower positive feedback during COVID-19 compared to previous years, implying that these services struggled to sustain patient satisfaction amid pandemic-related disruptions.

### Demographic disparities and health equity

Our examination of hospitals in areas with different percentages of visible minorities and low-income populations revealed clear differences in patient satisfaction patterns. From 2016 to 2020, facilities in regions with higher minority and low-income concentrations generally had better reviews, but this reversed in 2021–2022, with these same hospitals receiving considerably worse feedback. This abrupt shift may reflect the following structural imbalances:

**Resource gaps:** Financial constraints have worsened, as several Ontario hospitals face significant challenges balancing their budgets [74], limiting opportunities for expansions or improved services. Infrastructure shortfalls further compound the crisis, with many hospital buildings in poor condition and in need of repair [75]. The pandemic hit all hospitals hard, but those serving minority and low-income areas experienced especially acute pressures. These safety-net hospitals, already underfunded, lacked the capacity to handle surging demands and were forced to halt income-generating elective services, compounding their financial strain [76].

**Unequal health impacts:** Studies show that visible minority groups had higher rates of COVID-19 infection, hospitalization, and mortality [77–80]. This likely fueled higher negative feedback from these communities.

**Socioeconomic pressures:** Crowded housing, job instability, and limited preventive care made low-income communities more vulnerable to COVID-19 [81]. Hospitals in these areas faced chronic resource shortages, further stretching staff and supply chains [82,83].

**Language barriers:** Non-English speakers faced substantial communication challenges. One U.S. study found a two-fold increase in COVID-19 hospitalizations and over two times higher risk of death among non-English speakers [84].

**Trust issues:** Historical mistreatment of minority communities contributed to vaccine hesitancy and reluctance to seek care. Statistics Canada found that only 56.4% of the Black population was open to vaccination, compared to 77.7% of White and 82.5% of South Asian populations [85].

These findings point to the need for resilient healthcare systems that prioritize equity, particularly in times of crisis. Future work should identify robust care models for marginalized populations and explore how social determinants of health shape patient experiences.

### Implications for healthcare management and policy

**AI-enhanced pain management ecosystems:** An AI-based approach to pain management could transform patient care by using patient histories, treatment data, and real-time vitals to generate personalized pain relief strategies. This might help the 20% of Canadians who experience chronic pain and reduce the annual $38.2–$40.3 billion cost of pain management [86]. Yet, biases in AI algorithms, data privacy concerns, and the potential loss of human empathy remain real risks [87–89]. Emerging Canadian reports also convey the potential for rapid AI integration in healthcare settings could reshape patient-provider dynamics [90], while another recent study found that real-time AI alerts cut patient deaths by 43% [91].

**Integrated mental health frameworks:** Embedding mental health screening into regular medical workflows could address the uptick in ‘anxiety’ found in negative reviews from 2017 to 2022. With one in five Canadians facing mental illness each year, and around 4,000 annual suicides [92], building mental health support into hospital care could improve both physical and mental health outcomes.

**Predictive emergency care systems:** Advanced data analytics in emergency departments could improve patient flow and resource deployment. This strategy may counter the growing negative reviews in the Emergency unit, which rose from 36.1% in 2017 to 44.3% in 2021. Predictive models can forecast patient volumes with up to 90% accuracy [93], helping reduce wait times. Simulation-based methods and machine learning also show promise for identifying bottlenecks and providing real-time wait-time predictions [94,95], potentially increasing patient satisfaction in Canadian Emergency Departments (EDs), where 90% of non-admitted patients finish their visit within 9.1 hours [96]. Interestingly, after adopting SurgeCon, a platform that uses real-time analytics to monitor capacity and predict surges, Carbonear General Hospital in Newfoundland and Labrador cut physician wait times from 104 to 42 minutes and total ED time from 199 to 134 minutes [97].

**Blockchain-enabled billing transparency:** Blockchain, a decentralized digital ledger, provides real-time updates across a peer-to-peer network with tamper-resistant data integrity [98]. This technology is particularly suited to addressing healthcare billing challenges, where clarity and trust are paramount. Blockchain-based billing systems can provide transparent, immutable records, reducing disputes and enabling patients to better understand their medical expenses. This is especially critical given the significant patient dissatisfaction (68.34%) related to billing issues. Beyond transparency, the financial benefits are substantial. Research suggests that blockchain could save the healthcare industry $20 billion annually by streamlining data management and reducing inefficiencies [99]. Real-world implementations also showcase its potential. In 2019, Anthem, the second-largest health insurer in the U.S., announced plans to use blockchain to securely manage health data for 40 million patients [100]. Similarly, the United Arab Emirates (UAE) became the first nation to integrate blockchain and AI into its organ transplant system [101], further demonstrating the transformative potential of this technology.

**Recognition-centric care environments:** Environments that value staff recognition could boost patient satisfaction, reinforcing the 73.18% positive reviews tied to positive recognition during COVID-19. Studies suggest that better staff appreciation can lead to lower turnover rates and better patient safety [102,103]. However, the pandemic showed that recognition alone cannot solve deeper challenges like heavy workloads, stress, and inadequate pay [104,105]. For example, 90% of nurses reported increased workload since COVID-19 started, and 57% faced financial difficulties [105]. A comprehensive strategy that addresses these root issues is essential for lasting improvements in workforce morale and patient experiences.

**Adaptive care matrices for specialized units:** Flexible care frameworks in specialized areas such as dentistry or radiology could guard against service disruptions. Hospitals that shifted resources quickly during the pandemic saw fewer bottlenecks and better patient outcomes [106–110]. Implementing these strategies may prevent spikes in negative reviews when normal operations are disrupted.

**Health equity zone frameworks:** Targeted healthcare programs in high-minority regions might reverse the decline in positive feedback during the pandemic and honor Canada’s goal to reduce health inequalities. Research indicates that indigenous and low-income groups experience higher rates of mental illness and worse health outcomes [111], calling for policy interventions like equity zones to improve care and well-being [112,113].

Building on the interventions described above, putting them into practice will require strong privacy protections, regular performance reviews, and integration with existing hospital systems. For AI tools, whether in pain management, feedback dashboards, or predictive emergency care, this means secure data handling, safeguards against bias, and pilot testing in high-impact areas before wider rollout. Blockchain billing can be introduced in stages, starting with high-volume services to demonstrate transparency and train staff in secure processes. Interventions that focus on mental health, staff recognition, or flexible care models should be embedded into everyday workflows and supported by policies that address workload, resources, and equity. Health equity zone initiatives will benefit from targeted outreach, resource allocation, and partnerships with community organizations. Across all approaches, small-scale pilots and active involvement of staff and patients in design will help identify challenges early and improve adoption.

### Limitations

This study offers valuable insights but has certain limitations. First, the absence of healthcare provider perspectives limits our view of the factors that shape satisfaction, although our analysis of patient reviews across sentiment, clinical entities, and themes provides a broad perspective. Second, our lack of detailed demographic data on patient ethnicity and gender might introduce bias, so we utilized regional statistics on visible minorities and low-income populations to approximate healthcare equity. Third, not every patient leaves a review, and dissatisfied individuals are often more likely to do so, which may skew findings and lead to a selection bias.

Fourth, several contextual factors that we could not fully model may also shape patient sentiment. During early pandemic waves, many hospitals postponed elective procedures, limited visitor access, and expanded telemedicine, changes that could have altered both the volume and tone of reviews. Public campaigns that celebrated front-line workers may likewise have encouraged more favourable posts. Furthermore, online feedback platforms capture a self-selected subset of patients, often those with strong opinions or reliable internet access. Future work that links review text with administrative records or population surveys could help to quantify these influences and refine the estimates reported here.

Fifth, we removed incomplete data, potentially excluding some experiences, and ambiguous hospital unit names required approximate mappings, possibly introducing inaccuracies. Sixth, the dataset from 2015 is relatively small (about 500 reviews), which may limit robustness for that year, but it still revealed consistent trends. Seventh, we do not know which sites are teaching hospitals, a factor that can affect patient perceptions. Finally, without contextual information about each patient’s circumstances, we cannot fully dissect the roots of their feedback. However, we employed advanced natural language processing methods to extract deeper insights, partly offsetting this gap.

Despite these constraints, our findings establish a strong platform for future investigations. They spotlight key areas like staff interactions, billing transparency, and health equity, offering clear pathways for policy and practice improvements.

## Conclusion

This analysis of patient reviews indicates major gaps in hospital patient satisfaction before and during the COVID-19 pandemic, with many hospitals struggling to meet rising patient expectations. Staff behaviour stands out as a decisive factor, as kindness, professionalism, and helpfulness shape positive perceptions. Hospitals that invest in empathy, improved communication, and patient-focused care may see meaningful boosts in satisfaction. The pandemic affected patient satisfaction unevenly across hospital units. Some units maintained higher positive ratings, while others faced recurring problems linked to anxiety and infection. The dip in negative reviews, along with a rise in positive ones, may suggest greater public empathy or improvements in service quality. However, this also illustrates how quickly patient expectations can shift during crises.

Regional disparities further complicate the picture, as satisfaction patterns switched course during the pandemic, stressing the importance of coordinated health equity measures. These findings offer a clear framework for hospitals aiming to refine patient experiences, by tightening communication, resolving shortcomings in low-performing units, encouraging staff recognition, and tackling billing challenges. Interestingly, a recent Ontario-based study found that hospitals rarely shared patient feedback with the public, limiting opportunities for improvement [60]. Relying on ongoing analysis of patient reviews, supported by NLP techniques, can identify emerging concerns early and direct adjustments that keep pace with changing community needs.

## Supporting information

S1 Table Standardized hospital unit names. Standardized list of hospital unit names used for NLP analysis.
(PDF)

S2 Table Thematic satisfaction categories. Thematic satisfaction categories used in patient review classification.
(PDF)

S3 Table Full results of COVID × Unit logistic regression. Model summary, likelihood-ratio test, and complete coefficient estimates for *Sentiment* ∼ *CovidPeriod* × *Unit*.
(PDF)

S4 Table Logistic regression with CovidPeriod × ThemeCode interaction. Model summary, likelihood-ratio test, and full coefficients for *Sentiment* ∼ *CovidPeriod* × *ThemeCode*.
(PDF)

S5 Table Logistic regression with CovidPeriod × Minority interaction. Model summary and coefficient estimates for *Sentiment* ∼ *CovidPeriod* × *Minority*.
(PDF)

S6 Table Logistic regression with CovidPeriod × Low_income interaction. Model summary and coefficient estimates for *Sentiment* ∼ *CovidPeriod* × *Low_income*.
(PDF)
