# Supplementary material for: From patient voices to policy: Data analytics reveals patterns in Ontario’s hospital feedback
Source: PLOS Digit Health. 2026 Feb 5;5(2):e0000739. doi: 10.1371/journal.pdig.0000739 (PMC12875584; doi:10.1371/journal.pdig.0000739)
Supplement: S1 Table — Standardized list of hospital unit names used for NLP analysis. (PDF) [file pdig.0000739.s001.pdf]

S1 Table. Standardized Hospital Unit Names

Table S1. Standardized list of hospital unit names used for NLP analysis

Unit ID	Hospital Unit Name
1	Emergency Department / Accident and Emergency (A&E)
2	Intensive Care (ICU) / Critical Care
3	Coronary Care (CCU) / Cardiac Intensive Care (CICU)
4	Burn / Burn Center
5	Cardiology
6	Radiology
7	Orthopedics
8	Geriatrics
9	Pediatrics
10	Psychiatry
11	Obstetrics
12	Urology
13	Gynecology
14	Postpartum
15	Ophthalmology
16	Dialysis (Renal)
17	Oncology / Cancer Center
18	Neurology
19	Rehabilitation
20	Day Surgery
21	Palliative Care
22	Respiratory
23	Pre-admission Clinic (PAC)
24	Inpatient Ward
25	Outpatient
26	Mental Health
27	Medicine
