# Supplementary material for: From patient voices to policy: Data analytics reveals patterns in Ontario’s hospital feedback
Source: PLOS Digit Health. 2026 Feb 5;5(2):e0000739. doi: 10.1371/journal.pdig.0000739 (PMC12875584; doi:10.1371/journal.pdig.0000739)
Supplement: S2 Table — Thematic satisfaction categories used in patient review classification. (PDF) [file pdig.0000739.s002.pdf]

S2 Table. Thematic Satisfaction Categories

Table S2. Thematic satisfaction categories used in patient review classification

**Theme ID**	**Theme Label**
1	nurse/nurse aide
2	respect to patient
3	dietary/service
4	information/education
5	families/friends
6	housekeeping/room
7	access/coord of care
8	emergency
9	general comment
10	physical comfort
11	emotional support
12	infection prevention & control
13	parking/transport
14	medication/prescription
15	discharge
16	billing/accounting
17	admit/registration
18	religion
19	continuity/transition
20	positive recognition
21	radiology
22	cardiology
23	icu/ccu
24	laboratory
25	social services
26	met- access/coord of care
