# Supplementary material for: From patient voices to policy: Data analytics reveals patterns in Ontario’s hospital feedback
Source: PLOS Digit Health. 2026 Feb 5;5(2):e0000739. doi: 10.1371/journal.pdig.0000739 (PMC12875584; doi:10.1371/journal.pdig.0000739)
Supplement: S3 Table — Model summary, likelihood-ratio test, and complete coefficient estimates for Sentiment ∼ CovidPeriod × Unit. (PDF) [file pdig.0000739.s003.pdf]

S3 Table. Full Results of COVID × Unit Logistic Regression

Model Summary

- **Log-likelihood:** –42,552
- **Pseudo R^2:** 0.0205 (Cox–Snell)
- **Method:** Iteratively Reweighted Least Squares (IRLS)
- **Iterations:** 5

Likelihood Ratio Test (Interaction vs. No Interaction)

LR Test: $\chi^2 = 35.58$, df = 19, $p = 0.0119$

This result indicates a statistically significant interaction between unit type and COVID-period, suggesting that the effect of the pandemic on sentiment varied by unit.

Full Regression Coefficient Table

Table S3. Full logistic regression output: Sentiment $\sim$ CovidPeriod $\times$ Unit

**Term**	**Odds (95% CI)**	**p-value**
Intercept	1.35 (1.30–1.39)	< 0.001
CovidPeriod	1.38 (1.31–1.46)	< 0.001
Burn Unit / Burn Center	1.00 (1.00–1.00)	0.072
Cardiology Unit	1.28 (1.15–1.43)	< 0.001
CCU / CICU	2.33 (1.76–3.08)	<0.001
Day Surgery Unit	1.49 (1.32–1.69)	<0.001
Dentistry	2.28 (1.50–3.47)	<0.001
Dialysis (Renal) Unit	1.00 (1.00–1.00)	0.097
Emergency Department	0.68 (0.64–0.71)	<0.001
Gynecology Unit	1.44 (0.95–2.19)	0.083
Medicine Unit	0.67 (0.62–0.73)	<0.001
Mental Health	0.60 (0.46–0.78)	<0.001
Neurology Unit	0.92 (0.71–1.18)	0.514
Obstetrics Unit	1.02 (0.90–1.15)	0.775
Oncology / Cancer Center	0.89 (0.79–1.00)	0.058
Ophthalmology Unit	1.15 (0.90–1.48)	0.268
Orthopedics Unit	0.86 (0.75–0.99)	0.038
Outpatient	1.45 (1.31–1.60)	<0.001
Pediatrics Unit	0.97 (0.83–1.13)	0.702
Radiology Unit	1.33 (0.90–1.97)	0.150
Rehabilitation Unit	0.99 (0.83–1.18)	0.872
Respiratory Unit	1.25 (0.84–1.87)	0.274
Urology Unit	1.14 (0.89–1.45)	0.305
Burn Unit / Burn Center_x_Covid	1.0000 (1.0000–1.0000)	0.364
Cardiology Unit_x_Covid	0.9311 (0.7799–1.1104)	0.438
CCU / CICU_x_Covid	0.8327 (0.4244–1.6347)	0.596
Day Surgery Unit_x_Covid	1.2760 (1.0501–1.5493)	0.016
Dentistry_x_Covid	0.4140 (0.2169–0.7894)	0.008
Dialysis (Renal) Unit_x_Covid	1.0000 (1.0000–1.0000)	0.013
Emergency Department_x_Covid	1.0214 (0.9423–1.1065)	0.602
Gynecology Unit_x_Covid	1.2096 (0.4939–2.9624)	0.677
Medicine Unit_x_Covid	1.0854 (0.9556–1.2335)	0.204
Mental Health_x_Covid	0.8884 (0.6020–1.3108)	0.552
Neurology Unit_x_Covid	0.8514 (0.5161–1.4047)	0.529
Obstetrics Unit_x_Covid	1.0990 (0.8992–1.3430)	0.370
Oncology / Cancer Center_x_Covid	1.4569 (1.1027–1.9251)	0.008
Ophthalmology Unit_x_Covid	1.1744 (0.7932–1.7387)	0.420
Orthopedics Unit_x_Covid	0.8990 (0.6790–1.1895)	0.462
Outpatient_x_Covid	0.8325 (0.6597–1.0509)	0.125
Pediatrics Unit_x_Covid	1.0214 (0.7844–1.3298)	0.878
Radiology Unit_x_Covid	0.6039 (0.3273–1.1143)	0.104
Rehabilitation Unit_x_Covid	1.2420 (0.9329–1.6520)	0.140
Respiratory Unit_x_Covid	1.3201 (0.6265–2.7843)	0.465
Urology Unit_x_Covid	0.7695 (0.5097–1.1626)	0.208
