# Supplementary material for: From patient voices to policy: Data analytics reveals patterns in Ontario’s hospital feedback
Source: PLOS Digit Health. 2026 Feb 5;5(2):e0000739. doi: 10.1371/journal.pdig.0000739 (PMC12875584; doi:10.1371/journal.pdig.0000739)
Supplement: S4 Table — Model summary, likelihood-ratio test, and full coefficients for Sentiment ∼ CovidPeriod × ThemeCode. (PDF) [file pdig.0000739.s004.pdf]

# S4 Table. Logistic Regression with CovidPeriod × ThemeCode Interaction

**Model Summary:**

- Observations: 129,858
- Method: Iteratively Reweighted Least Squares (IRLS)
- Log-Likelihood: -71,840
- Null Log-Likelihood: -89,975
- Pearson Chi-Square: 130,000
- Pseudo $R^2$: 0.202

**Likelihood-Ratio Test for Interaction:**

LR test for CovidPeriod × ThemeCode: $\chi^2 = 159.40$, df = 25, $p < 0.0001$

**Table S4.** Full logistic regression results: Sentiment $\sim$ CovidPeriod $\times$ ThemeCode

| **Variable** | **Odds (95% CI)** | ***p*-value** |
|---|---|---|
| Intercept | 0.34 [0.32–0.35] | $< 0.001$ |
| CovidPeriod | 1.41 [1.32–1.50] | $< 0.001$ |
| Theme admit/registration | 1.12 [0.98–1.27] | 0.0902 |
| Theme billing/accounting | 0.15 [0.09–0.24] | $< 0.001$ |
| Theme cardiology | 4.27 [3.51–5.18] | $< 0.001$ |
| Theme continuity/transition | 1.55 [1.33–1.80] | $< 0.001$ |
| Theme dietary/service | 0.86 [0.77–0.96] | 0.0063 |
| Theme discharge | 0.33 [0.27–0.41] | $< 0.001$ |
| Theme emergency | 1.71 [1.60–1.84] | $< 0.001$ |
| Theme emotional support | 6.93 [6.38–7.54] | $< 0.001$ |
| Theme families/friends | 0.97 [0.82–1.15] | 0.7231 |
| Theme general comment | 47.70 [43.17–52.71] | $< 0.001$ |
| Theme housekeeping/room | 1.02 [0.91–1.13] | 0.7813 |
| Theme icu/ccu | 6.03 [4.88–7.46] | $< 0.001$ |
| Theme infection prevention & control | 0.41 [0.30–0.56] | $< 0.001$ |
| Theme information/education | 1.47 [1.35–1.59] | $< 0.001$ |
| Theme laboratory | 0.95 [0.72–1.25] | 0.7094 |
| Theme medication/prescription | 0.41 [0.35–0.48] | $< 0.001$ |
| Theme met- access/coord of care | 0.00 [0.00–inf] | 0.9993 |
| Theme nurse/nurse aide | 4.14 [3.91–4.39] | $< 0.001$ |
| Theme parking/transport | 0.41 [0.30–0.56] | $< 0.001$ |
| Theme physical comfort | 2.37 [2.25–2.49] | $< 0.001$ |
| Theme positive recognition | 933.56 [465.91–1870.60] | $< 0.001$ |
| Theme radiology | 0.98 [0.85–1.15] | 0.8430 |
| Theme religion | 4.46 [2.95–6.73] | $< 0.001$ |
| Theme respect to patient | 8.19 [7.69–8.72] | $< 0.001$ |
| Theme social services | 1.68 [1.17–2.41] | 0.0046 |
| Theme admit/registration_x_Covid | 0.94 [0.75–1.16] | 0.5484 |
| Theme billing/accounting_x_Covid | 1.24 [0.58–2.62] | 0.5800 |
| Theme cardiology_x_Covid | 0.71 [0.52–0.99] | 0.0418 |
| Theme continuity/transition_x_Covid | 0.74 [0.59–0.93] | 0.0088 |
| Theme dietary/service_x_Covid | 0.77 [0.64–0.92] | 0.0032 |
| Theme discharge_x_Covid | 0.69 [0.47–1.00] | 0.0476 |
| Theme emergency_x_Covid | 1.03 [0.92–1.16] | 0.5757 |
| Theme emotional support_x_Covid | 0.73 [0.64–0.83] | <0.001 |
| Theme families/friends_x_Covid | 0.58 [0.45–0.75] | <0.001 |
| Theme general comment_x_Covid | 0.66 [0.57–0.76] | <0.001 |
| Theme housekeeping/room_x_Covid | 1.28 [1.08–1.52] | 0.0042 |
| Theme icu/ccu_x_Covid | 0.65 [0.46–0.92] | 0.0145 |
| Theme infection prevention & control_x_Covid | 2.32 [1.59–3.38] | <0.001 |
| Theme information/education_x_Covid | 0.72 [0.63–0.81] | <0.001 |
| Theme laboratory_x_Covid | 0.79 [0.51–1.23] | 0.2965 |
| Theme medication/prescription_x_Covid | 0.89 [0.69–1.14] | 0.3624 |
| Theme met- access/coord of care_x_Covid | $\infty$ [0.00–$\infty$] | 0.9990 |
| Theme nurse/nurse aide_x_Covid | 0.90 [0.82–0.98] | 0.0226 |
| Theme parking/transport_x_Covid | 1.35 [0.79–2.30] | 0.2690 |
| Theme physical comfort_x_Covid | 0.88 [0.81–0.96] | 0.0022 |
| Theme positive recognition_x_Covid | 0.73 [0.22–2.43] | 0.6092 |
| Theme radiology_x_Covid | 0.71 [0.56–0.90] | 0.0047 |
| Theme religion_x_Covid | 0.71 [0.33–1.51] | 0.3749 |
| Theme respect to patient_x_Covid | 0.78 [0.71–0.87] | <0.001 |
| Theme social services_x_Covid | 0.90 [0.51–1.60] | 0.7183 |
