# Supplementary material for: From patient voices to policy: Data analytics reveals patterns in Ontario’s hospital feedback
Source: PLOS Digit Health. 2026 Feb 5;5(2):e0000739. doi: 10.1371/journal.pdig.0000739 (PMC12875584; doi:10.1371/journal.pdig.0000739)
Supplement: S5 Table — Model summary and coefficient estimates for Sentiment ∼ CovidPeriod × Minority. (PDF) [file pdig.0000739.s005.pdf]

S5 Table. Logistic Regression with CovidPeriod × Minority Interaction

Model Summary:

- Observations: 63,592
- Method: MLE
- Log-Likelihood: -43,091
- Null Log-Likelihood: -43,254
- Pseudo R^2: 0.003770

Likelihood-Ratio Test for Interaction:

LR test for CovidPeriod × ThemeCode: $\chi^2 = 3.73$, df = 1, p = 0.0536

Table S5. Logistic regression results: Sentiment ∼ CovidPeriod × Minority

**Variable**	**Odds (95% CI)**	p**-value**
Intercept	1.25 [1.22—1.28]	< 0.001
Minority (True)	0.97 [0.93—1.01]	0.171
CovidPeriod	1.37 [1.31—1.43]	< 0.001
CovidPeriod × Minority (True)	0.94 [0.88—1.00]	0.053
