# Supplementary material for: From patient voices to policy: Data analytics reveals patterns in Ontario’s hospital feedback
Source: PLOS Digit Health. 2026 Feb 5;5(2):e0000739. doi: 10.1371/journal.pdig.0000739 (PMC12875584; doi:10.1371/journal.pdig.0000739)
Supplement: S6 Table — Model summary and coefficient estimates for Sentiment ∼ CovidPeriod × Low_income. (PDF) [file pdig.0000739.s006.pdf]

S6 Table. Logistic Regression with CovidPeriod × Low-income Interaction

Model Summary:

- Observations: 63,592
- Method: MLE
- Log-Likelihood: -43,063
- Null Log-Likelihood: -43,254
- Pseudo R^2: 0.004421

Likelihood-Ratio Test for Interaction:

LR test for CovidPeriod × ThemeCode: $\chi^2 = 24.86$, df = 1, $p < 0.001$

Table S6. Logistic regression results: Sentiment ∼ CovidPeriod × Low_income

**Variable**	**Odds (95% CI)**	***p*-value**
Intercept	1.15 [1.12—1.18]	< 0.001
Low_income[T.True]	1.19 [1.14—1.24]	< 0.001
CovidPeriod	1.44 [1.38—1.50]	< 0.001
CovidPeriod:Low_income[T.True]	0.84 [0.79—0.90]	< 0.001
